# The Interaction of *N*-Acylhomoserine Lactone Quorum Sensing Signaling Molecules with Biological Membranes: Implications for Inter-Kingdom Signaling

**DOI:** 10.1371/journal.pone.0013522

**Published:** 2010-10-20

**Authors:** Benjamin Michael Davis, Rasmus Jensen, Paul Williams, Paul O'Shea

**Affiliations:** 1 Cell Biophysics Group, Institute of Biophysics, Imaging and Optical Science, University of Nottingham, Nottingham, United Kingdom; 2 School of Molecular Medical Sciences, Centre for Biomolecular Sciences, University of Nottingham, Nottingham, United Kingdom; East Carolina University School of Medicine, United States of America

## Abstract

**Background:**

The long chain *N*-acylhomoserine lactone (AHL) quorum sensing signal molecules released by *Pseudomonas aeruginosa* have long been known to elicit immunomodulatory effects through a process termed inter-kingdom signaling. However, to date very little is known regarding the exact mechanism of action of these compounds on their eukaryotic targets.

**Methodology/Principal Findings:**

The use of the membrane dipole fluorescent sensor di-8-ANEPPS to characterise the interactions of AHL quorum sensing signal molecules, *N*-(3-oxotetradecanoyl)-L-homoserine lactone (3-oxo-C14-HSL), *N*-(3-oxododecanoyl)homoserine-L-lactone (3-oxo-C12-HSL) and *N*-(3-oxodecanoyl) homoserine-L-lactone (3-oxo-C10 HSL) produced by *Pseudomonas aeruginosa* with model and cellular membranes is reported. The interactions of these AHLs with artificial membranes reveal that each of the compounds is capable of membrane interaction in the micromolar concentration range causing significant modulation of the membrane dipole potential. These interactions fit simple hyperbolic binding models with membrane affinity increasing with acyl chain length. Similar results were obtained with T-lymphocytes providing the evidence that AHLs are capable of direct interaction with the plasma membrane. 3-oxo-C12-HSL interacts with lymphocytes via a cooperative binding model therefore implying the existence of an AHL membrane receptor. The role of cholesterol in the interactions of AHLs with membranes, the significance of modulating cellular dipole potential for receptor conformation and the implications for immune modulation are discussed.

**Conclusions/ Significance:**

Our observations support previous findings that increasing AHL lipophilicity increases the immunomodulatory activity of these quorum compounds, while providing evidence to suggest membrane interaction plays an important role in quorum sensing and implies a role for membrane microdomains in this process. Finally, our results suggest the existence of a eukaryotic membrane-located system that acts as an AHL receptor.

## Introduction

Quorum sensing (QS) is the process through which bacterial cells communicate enabling unicellular populations to coordinate their response to an external stimulus as a function of population density, for a review see [1]. Gram negative bacteria such as *Pseudomonas aeruginosa* employ *N*-acylhomoserine lactone (AHL) QS signal molecules. *P. aeruginosa* is an opportunistic human pathogen responsible for causing infection in immune compromised individuals and is the leading cause of morbidity and mortality in cystic fibrosis patients [2].

**Figure 1:**
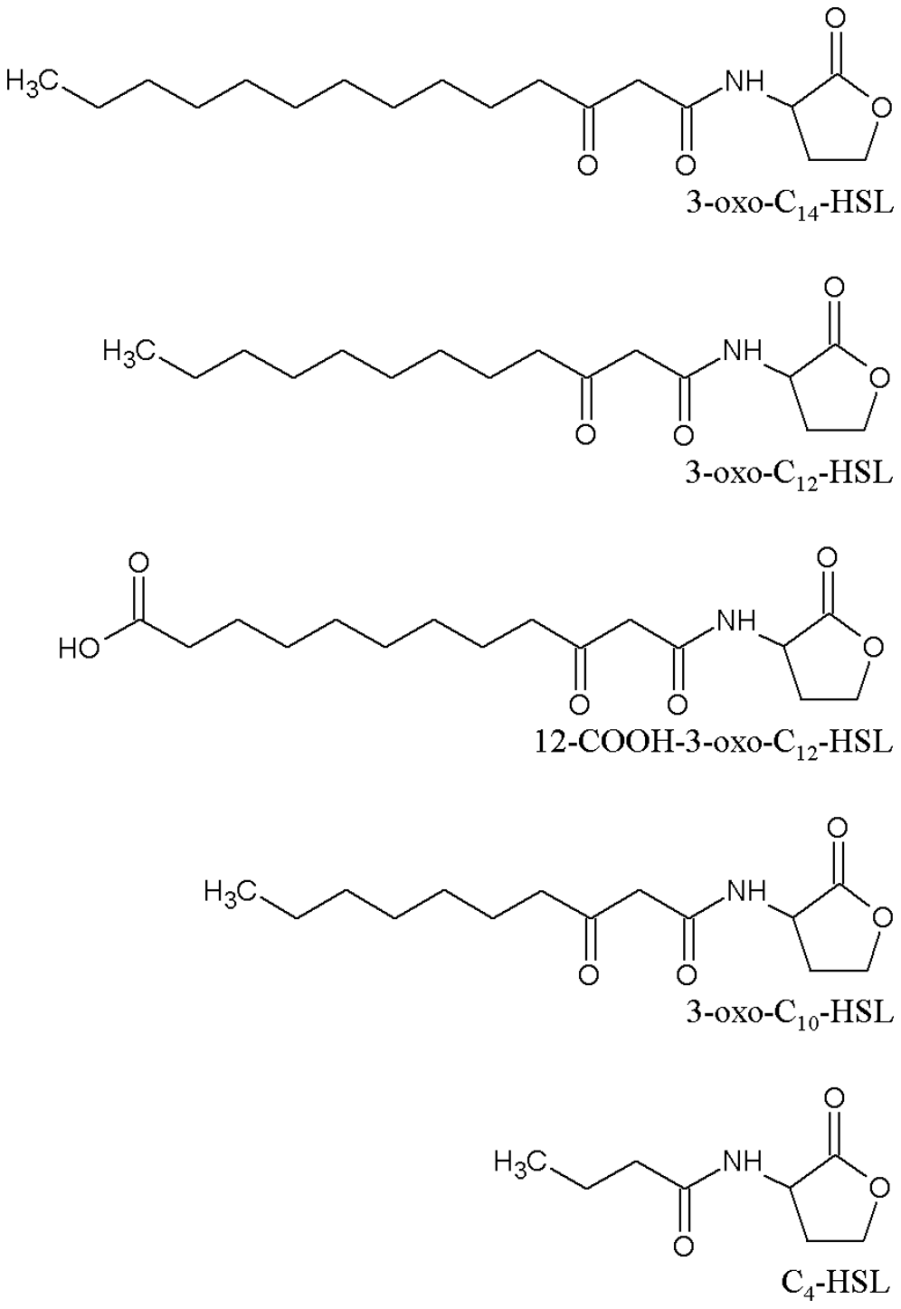
Structures of common AHLs synthesized by *P aeruginosa.* Structures of the most common AHL quorum sensing compounds synthesized by *Pseudomonas aeruginosa* (C_4_-HSL, and 3-oxo-C_12_-HSL) and those used in this study (3-oxo-C_14_-HSL, 3-oxo-C_12_-HSL, COOH-3-oxo-C_12_-HSL and 3-oxo-C_10_-HSL).

*P. aeruginosa* employs an AHL-dependent QS system employing two LuxR/I pairs (LasR/I and RhlR/I) where LasR and RhlR are transcriptional regulators which respond to the AHLs, *N*-(3-oxododecanoyl)homoserine lactone (3-oxo-C_12_-HSL) and *N*-butanoylhomoserine lactone (C_4_-HSL) (Figure 1) respectively. These are produced via the LasI and RhlI AHL synthases respectively [3]. Although LasI directs the synthesis primarily of 3-oxo-C_12_-HSL, the analogues 3-oxo-C_10_-HSL and 3-oxo-C_14_-HSL are also produced, albeit at much lower levels [4]. The *las* and *rhl* systems directly or indirectly regulate over 10% of the *P. aeruginosa* genome [5] and are organized as a hierarchy in which LasR/3-oxo-C_12_-HSL drives the expression of *lasI* (so constituting a positive feedback loop) as well as *rhlR* and *rhlI* [6]. The *las/rhl* QS system plays a key role in controlling virulence factor production, biofilm maturation, swarming motility and the expression of antibiotic efflux pumps [3].

*P. aeruginosa* AHLs have been detected *in vivo* during human infections. They are readily detectable in sputum from cystic fibrosis patients [7] although determining their physiological QS concentration range is complicated as a consequence of their susceptibility to alkaline [8] and/or enzymatic hydrolysis [9]. Apart from modulating bacterial gene expression, AHLs such as 3-oxo-C_12_-HSL (but not C_4_-HSL) antagonize growth and virulence factor production in Gram positive bacteria such as *Staphylococcus aureus* [10]. 3-oxo-C_12_-HSL may also contribute directly to the outcome of host-pathogen interactions. 3-oxo-C_12_-HSL influences smooth muscle contraction in blood vessels, exerts a marked bradycardia [11] and modulates the junctional integrity and paracellular permeability of epithelial cells [12]. It also modulates host inflammatory and immune responses, (reviewed in [13], [14]). For example, at concentrations below 10 µM, 3-oxo-C_12_-HSL reduced lipopolysaccharide (LPS)-induced production of the pro-inflammatory cytokine IL-12 by monocytes [15] whereas pro-inflammatory or pro-apoptotic effects were apparent at higher concentrations in both macrophages and neutrophils [14], [16], [17]. The proliferation and function (cytokine production) of both mitogen-stimulated (e.g., [18], [19] and antigen-stimulated [20]) T lymphocytes as well as antibody production by B lymphocytes [15], [19] are inhibited by 3-oxo-C_12_-HSL. Smith *et al.* [16] reported that 3-oxo-C_12_-HSL induced activation of the pro-inflammatory signaling components Cox-2 and NF-κB in transformed cell lines however, this does not occur in primary cells in the absence of LPS neither does 3-oxo-C_12_-HSL act via known pathogen pattern recognition receptors [21]. In the absence of LPS, 3-oxo-C_12_-HSL does induce phosphorylation of mitogen-activated protein kinase (MAPK) p38, which could modulate cytokine production and also potentiate TNFα-induced poly(adenosine 5′-diphosphate-ribose) (PARP) cleavage, a biochemical marker of apoptosis [22].

The direct target(s) of 3-oxo-C_12_-HSL in mammalian cells have yet to be fully characterized. Since 3-oxo-C_12_-HSL enters mammalian cells and retains intracellular activity [23], [24], the most likely receptor(s) for 3-oxo-C_12_-HSL has been suggested to be intracellular. In this context, Jahoor *et al.* [25] obtained evidence to suggest that 3-oxo-C_12_-HSL may bind to at least two isoforms (PPARγ and PPARβ/δ) of the peroxisome proliferator activated receptors (PPARs). These belong to the nuclear hormone receptor family which bind a range of endogenous and exogenous lipids and play roles in inflammation and lipid metabolism. 3-oxo-C_12_-HSL may therefore modulate NF-κB signaling via the direct interaction with PPARs. Using an affinity matrix, Seabra et al. [26] identified calprotectin as a target although this calcium binding protein is unlikely to be the primary receptor for 3-oxo-C_12_-HSL. In order for QS signal molecules to interact with intracellular components they must first interact with the cell membranes. Structure-activity assays of 3-oxo-C_12_-HSL have revealed that optimal immune modulatory activity in a mouse splenocyte proliferation assay in common with QS in *P. aeruginosa* requires a C_11_ to C_13_ acyl chain, an intact homoserine lactone ring and L-configuration at the chiral centre, suggesting lipophilicity is important for QS immunosuppressive activity [18]. Based on these observations and given the broad biological activity of 3-oxo-C_12_-HSL in both pathogens and eukaryotic cells (particularly its action on leukocytes), we have explored the interactions of long chain AHLs with simple membranes and T lymphocytes. The potential interactions of QS signal molecules with many types of membrane have so far largely been ignored, although a recent study by Lowery *et al.* [27] indicated they may have effects on bacterial membrane permeability. This study presents evidence that 3-oxo-C_12_ HSL and two close structural analogues, 3-oxo-C_10_-HSL and 3-oxo-C_14_-HSL are capable of insertion into the lipid bilayer in both artificial membrane and Jurkat T-lymphocyte cell systems. Although the AHL concentrations used are higher than those reported in some human host samples e.g. sputum from cystic fibrosis patients [7], the latter are likely to be an underestimate since local AHL concentrations up to 600 µM have been detected in culture supernatants of *P. aeruginosa* biofilms grown *in vitro* [4].

In order to determine whether these compounds are capable of directly interacting with membranes they were studied using the fluorescent probe di-8-ANEPPS (e.g. [28], [29]). This technique yields binding information such as affinity and overall binding capacity as well as whether addition of AHLs impacts on the membrane organization. The roles of QS acyl chain length (C_10_ to C_14_) and cholesterol in membrane interactions are also investigated and the potential roles of membrane microdomains (or rafts) in immune modulation are discussed.

## Results

### AHL Immunosuppressive Activity is Dependent on membrane affinity (Lipophilicity)

**Figure 2:**
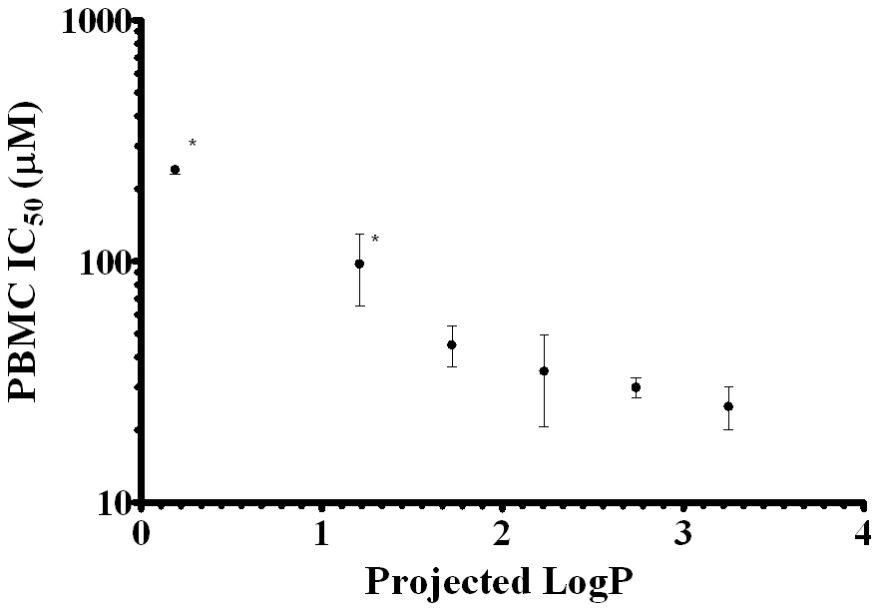
A strong negative correlation is observed between peripheral mononuclear cell proliferation and AHL lipophilicity. The IC_50_ for human peripheral mononuclear cell proliferation (PBMCs) isolated from three donors (* denotes n = 2) in the presence of AHLs of chain lengths between 8 and 14 carbons (values obtained from [18]) exhibit a strong negative correlation (Pearsons r = −0.895, *p* = 0.016) against the predicted octanol/water partition coefficient (LogP) for each compound (calculated using ACD/i-Lab LogP algorithm 12.0). n = 3, ±SEM. Projected LogP are given in Table 1.

The concentrations of AHLs required to inhibit 50% proliferation (IC_50_) of human peripheral blood mononuclear cells (PBMCs) was obtained from Chhabra et al 2003 [18]. These data are plotted against a calculated LogP value in Table 1, (calculated using the ACD/I-Lab Web service (ACD/LogP 12.0)) based on molecular structure. LogP is a measure of molecular lipophilicity and is frequently used in the pharmaceutical industry as an indicator of the likely bioavailability of a drug molecule. Figure 2 indicates that a strong negative correlation (Pearsons r = −0.895, *p* = 0.016) exists between membrane affinity and immunosuppressive activity.

**Table 1:** Comparison of AHL octanol/water partition coefficients.

QS molecule	Projected LogP (±0.49)
3-oxo-C14-HSL	3.25
3-oxo-C13 HSL	2.74
3-oxo-C12-HSL	2.23
3-oxo-C11-HSL	1.72
3-oxo-C10-HSL	1.21
3-oxo-C8-HSL	0.19
COOH-3-oxo-C12-HSL	0.18

Comparison of AHL octanol/water partition coefficients (LogP) values calculated from molecular structure using ACD/I-Lab LogP algorithm 12.0. Lipophilicity is observed to significantly increase as AHL chain length increases from C8 to C14 (r = 1.000, p<0.01, Spearman correlation). Addition of a carboxylic acid group to the 3-oxo-C12 HSL acyl chain results in a marked reduction in projected LogP. This compound was therefore expected to interact with membranes to a lesser degree than the major AHL released by *P. aeruginosa* 3-oxo-C12 HSL. LogP±95% confidence interval shown.

The observation that AHLs with increasing chain length (and therefore lipophilicity) impede cell proliferation to a greater extent provides the first fully quantitative evidence to suggest that the hydrophobic properties of AHLs play an important role in their ability to inhibit immune cell function. This will most likely manifest as an elevated ability to interact with biological membranes, influencing membrane electrical potentials which have been previously reported to effect indirectly membrane protein function [29]. Alternatively AHLs with greater lipophilicity will interact more readily with any hydrophobic binding pockets that exist within receptor systems (i.e. either in a membrane or in a soluble protein). The aim of this investigation is to provide evidence which of these mechanisms AHLs elicit their well documented immunomodulatory effects.

### Interactions of AHLs with Phospholipid Membrane Vesicles; Effects on Membrane Dipole Potential

Biological membranes, in addition to the well documented transmembrane potential also possess two lesser known electrical potentials termed the electrostatic membrane surface potential and the membrane dipole potential (for an extensive review of this trinity of membrane potentials see [30]). The membrane dipole potential has a magnitude in the region of 300 mV and arises from the orientation of dipoles at and just under the membrane surface. On insertion of a molecule of interest into biological membranes, this can cause a perturbation in this potential which can be detected in both artificial and cellular membrane systems using the electrochromic probe di-8-ANEPPS.

**Figure 3:**
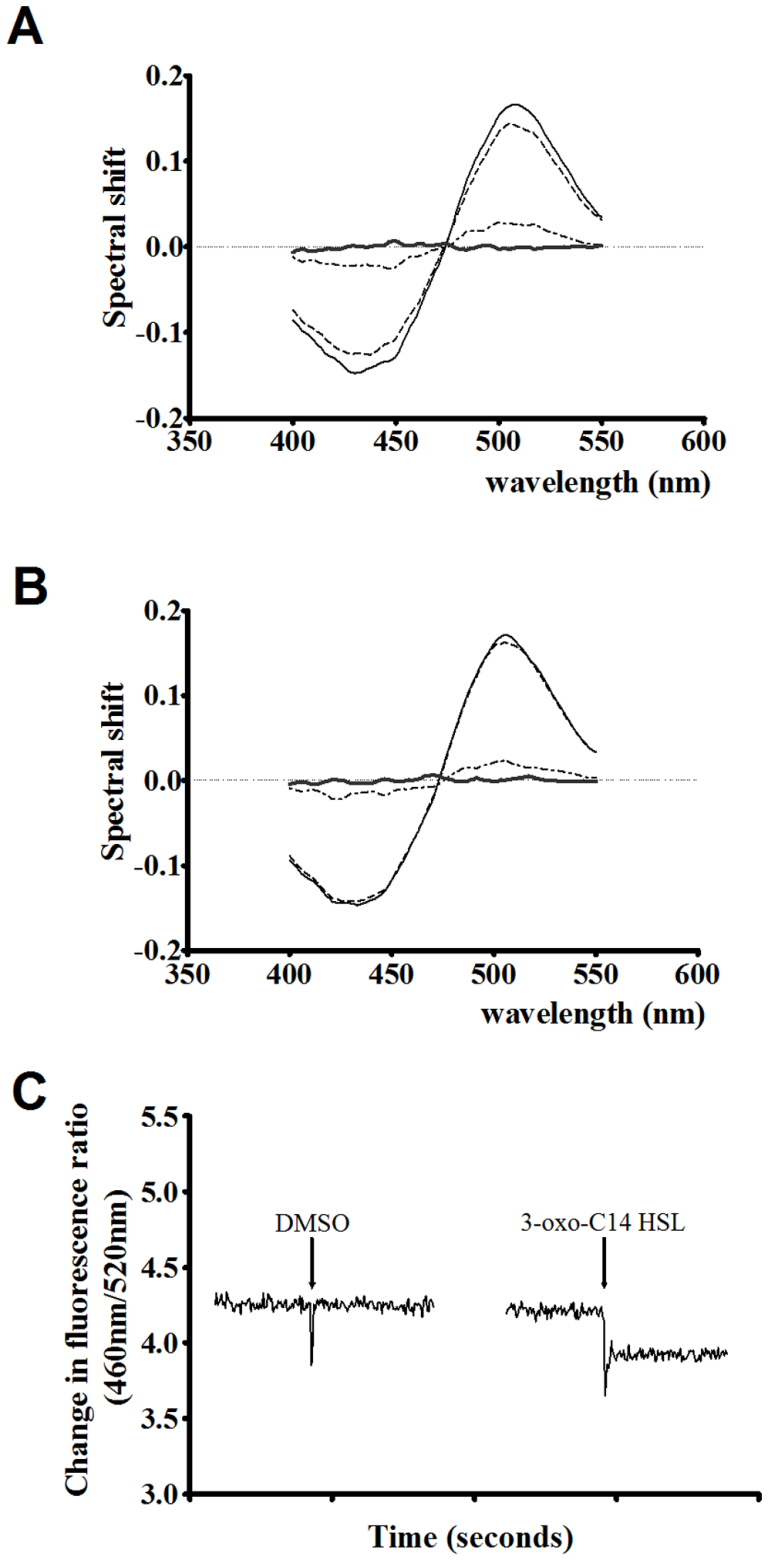
The interactions of AHLs with artificial membrane systems perturbs the membrane dipole potential. Fluorescence difference spectra obtained by subtracting di-8-ANEPPS excitation spectra (λem = 590 nm) of PC(100%) [A] or PC(70%)Cholesterol(30%) [B] membrane vesicles (400 µM) from those obtained after these membranes were exposed to the following QS molecules; 65 µM 3-oxo-C_14_-HSL (thick dashed line), 200 µM 3-oxo-C_12_-HSL (solid black line) and 200 µM 3-oxo-C_10_-HSL (thin dashed and dotted line). Before subtraction, each spectrum was normalized to the integrated areas so that the difference spectra would reflect only the spectral shifts. Each difference spectrum was then normalised to a DMSO control (grey line) and a three point moving average applied to reduce noise. In all experiments the dye concentration was 10 µM and temperature was maintained at 37°C. [C] A dual wavelength ratiometric measurement of the dipole potential variation in di-8-ANEPPS. Additions of 22 µM 3-oxo-C_14-_HSL or equivalent volumes of DMSO were made to 400 µM PC(100%). Samples were excited at 460 nm and 520 nm. Emission was read at 590 nm and the ratio *R*(460/520) was calculated (shown). All experiments n = 3.

On this basis it is shown in Figure 3A & B that addition of 3-oxo-C_14_-HSL, 3-oxo-C_12_-HSL and 3-oxo-C_10_-HSL to Phosphotidylcholine_100%_ and Phosphatidylcholine_70%_ Cholesterol_30%_ membranes vesicles led to a red shift in the excitation spectra of di-8-ANEPPS. A red shift (with a minimum of ∼440 nm and a maximum of ∼520 nm) is indicative of the ligands acting to decrease the membrane dipole potential upon insertion into the membrane. This finding is consistent with our previous work [29] reporting that addition of reagents known to decrease the membrane dipole potential, give rise to di-8-ANEPPS difference spectra of similar profiles to those reported here.

**Figure 4:**
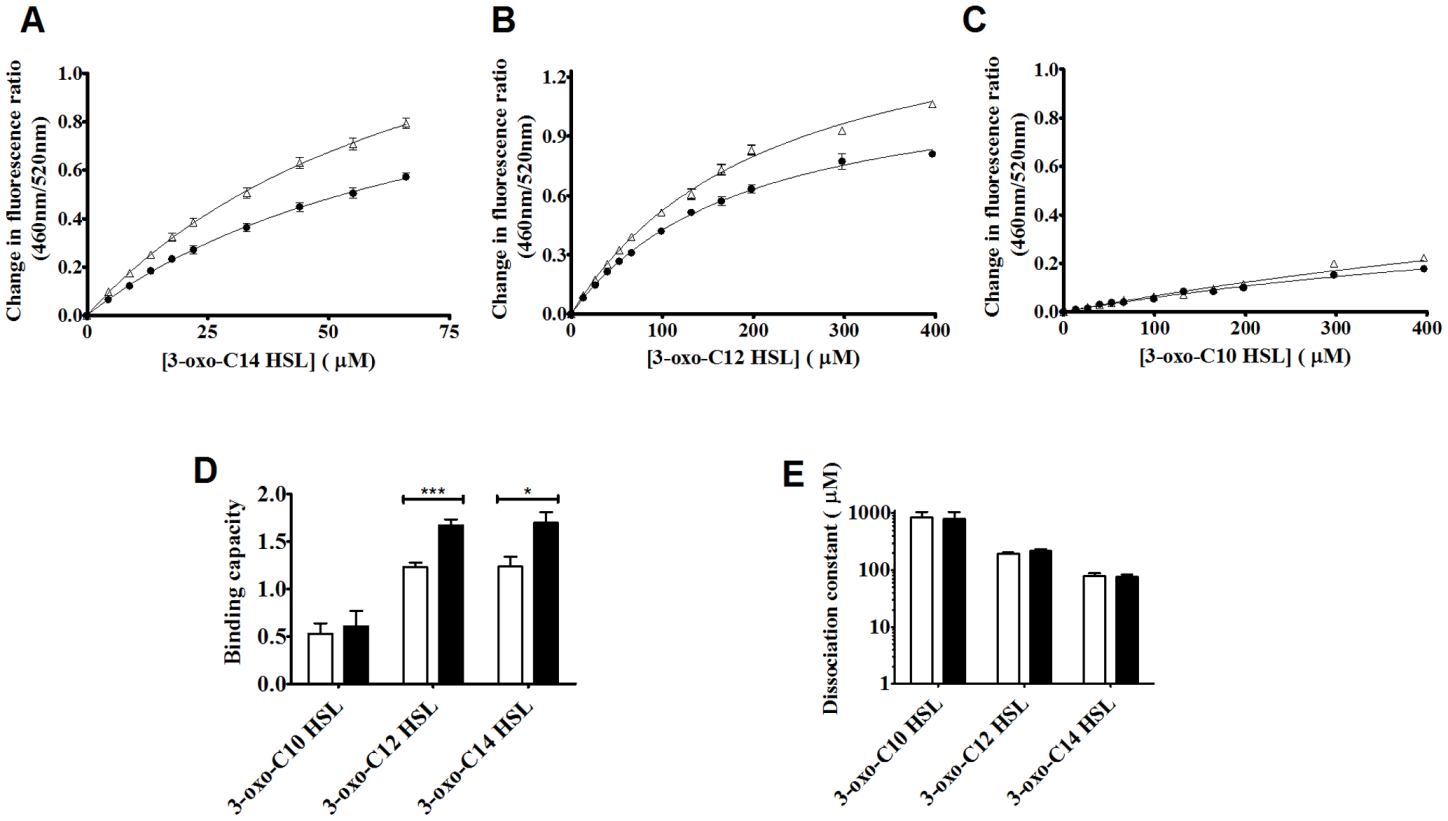
Binding profiles of the interactions of AHLs with artificial membrane systems. Binding profiles of [A] 3-oxo-C_14_-HSL, [B] 3-oxo-C_12_-HSL and [C] 3-oxo-C_10_-HSL on titration to PC_100%_ (•) or PC_70%_Cholesterol_30%_ (Δ) di-8-ANEPPS labeled liposomes (400 µM) at 37°C (n = 5) normalised to DMSO controls. Profiles were fitted to simple hyperbolic and sigmoidal binding models (equations 1 and 2) and extra sum of squares F-Tests were used to determine the best fitting in each case (all hyperbolic). [D] Average saturation points and [E] Average dissociation constant of the best fitting models±SEM.

Changes in the membrane dipole potential can be tracked over time using the ratio of di-8-ANEPPS fluorescence at 460 nm and 520 nm excitation and a fixed emission (termed R(460/520)), which is sensitive solely to variations of the local electric field due to dipolar molecular properties. Upon titration of the AHLs into these artificial membranes a concentration dependent decrease in membrane dipole potential was observed as shown in Figure 3C. These data were plotted as the incremental change of di-8-ANEPPS fluorescence versus the concentration of AHL added and fitted to various ‘binding’ models (eq. 1 & 2) as shown in Figure 4A–C. Such observations are consistent with the interaction and insertion of AHL molecules with the membrane vesicles. All ligands were found to interact with both Phosphotidylcholine_100%_ and Phosphatidylcholine_70%_Cholesterol_30%_ membranes via a simple hyperbolic (i.e. non-cooperative) binding mechanism, Figure 4D & E compare the dissociation constant (K_d_) and saturation point (B_max_) obtained in each case. Overall these figures depict that as acyl chain length increases it leads to a decrease of the observed the K_d_, suggesting that longer chain AHLs have a higher membrane affinity than the shorter chain variants. This was found to be the case for both Phosphotidylcholine_100%_ and Phosphatidylcholine_70%_Cholesterol_30%_ membranes. Titration of the AHLs into membranes containing 30% cholesterol did not result in a significant change in K_d_ (Figure 4D), however the binding capacity of 3-oxo-C_12_ HSL and 3-oxo-C_14_ HSL (i.e. saturation in fluorescence units) of Phosphatidylcholine_70%_ Cholesterol_30%_ membranes was significantly greater than for Phosphatidylcholine_100%_ membranes (p<0.05), suggesting that QS compounds may be accumulating in membrane microdomains present in Phosphatidylcholine_70%_Cholesterol_30%_ membranes.

**Figure 5:**
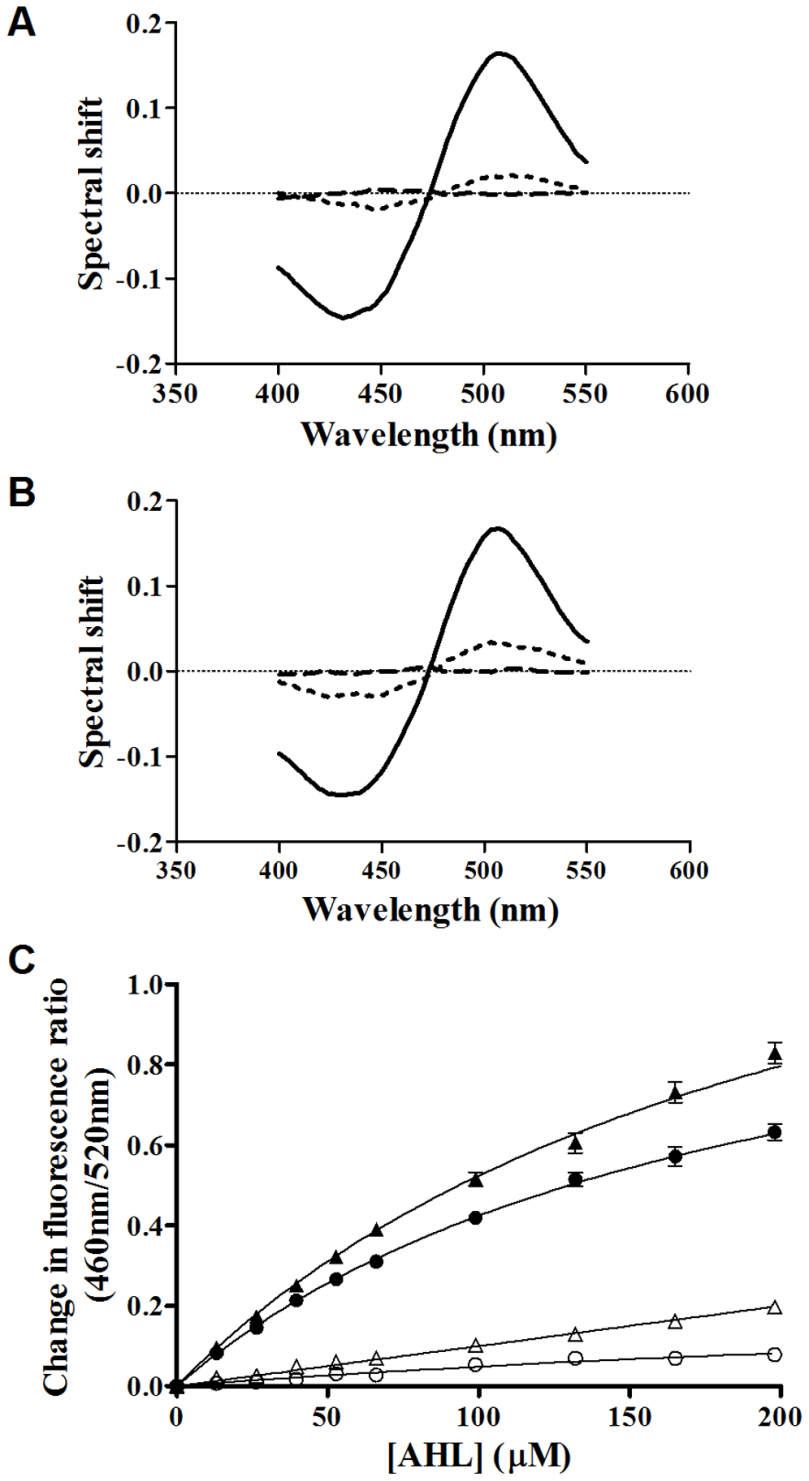
Comparing the interactions of COOH-3-oxo-C_12_ HSL and 3-oxo-C_12_ HSL with artificial membrane systems and their effects on membrane dipole potential. Fluorescence difference spectra obtained by subtracting di-8-ANEPPS excitation spectra (λem = 590 nm) of PC(100%) [A] or PC(70%)Cholesterol(30%) [B] membrane vesicles (400 µM) from those obtained after these membranes were exposed to either 200 µM 3-oxo-C_12_-HSL (dotted line, n = 5) or 200 µM COOH-3-oxo-C_12_-HSL (solid black line). Before subtraction, each spectrum was normalized to the integrated areas so that the difference spectra would reflect only the spectral shifts. Each difference spectrum was then normalised to a DMSO control (dashed line). In all experiments the dye concentration was 10 µM and temperature was maintained at 37°C. [C] Binding profiles of 3-oxo-C_12_ HSL (▴,•) and COOH-3-oxo-C_12_ HSL (Δ,○) on titration to PC_100%_ (circles) or PC_70%_Cholesterol_30%_ (triangles) di-8-anepps labeled liposomes (400 µM) at 37°C normalised to DMSO controls. Profiles were fitted to simple hyperbolic and sigmoidal binding models (equations 1 and 2) and extra sum of squares F-Tests were used to determine the best fitting in each case (3-oxo-C_12_ HSL was hyperbolic while COOH-3-oxo-C_12_ HSL fit poorly to both models). All experiments n = 3±SEM.

Addition of 200 µM COOH-3-oxo-C_12_ HSL to artificial membranes (Figure 5A & B) was found to cause only a nominal red shift in the di-8-anepps excitation spectrum. This shift was substantially less than that observed for 3-oxo-C_12_ HSL. Titration of COOH-3-oxo-C_12_ HSL into liposomes (Figure 5C) exhibited a dramatically lower effect on the dipole potential than 3-oxo-C_12_ HSL and fit poorly to both hyperbolic and sigmoidal models. This observation was expected as COOH-3-oxo-C_12_ HSL has a projected LogP substantially lower than 3-oxo-C_12_ HSL (Table 1).

### Interactions of AHLs with T-Lymphocytes; Effects on Membrane Dipole Potential

In the previous section it was shown that addition of AHLs to simple phospholipid membranes resulted in a decrease in the membrane dipole potential, which is indicative of these compounds inserting into artificial membranes. As a result, it was of interest to study their interactions with T-Lymphocytes which we utilize as a model for the interaction of quorum molecules with their prospective eukaryotic host cell systems.

**Figure 6:**
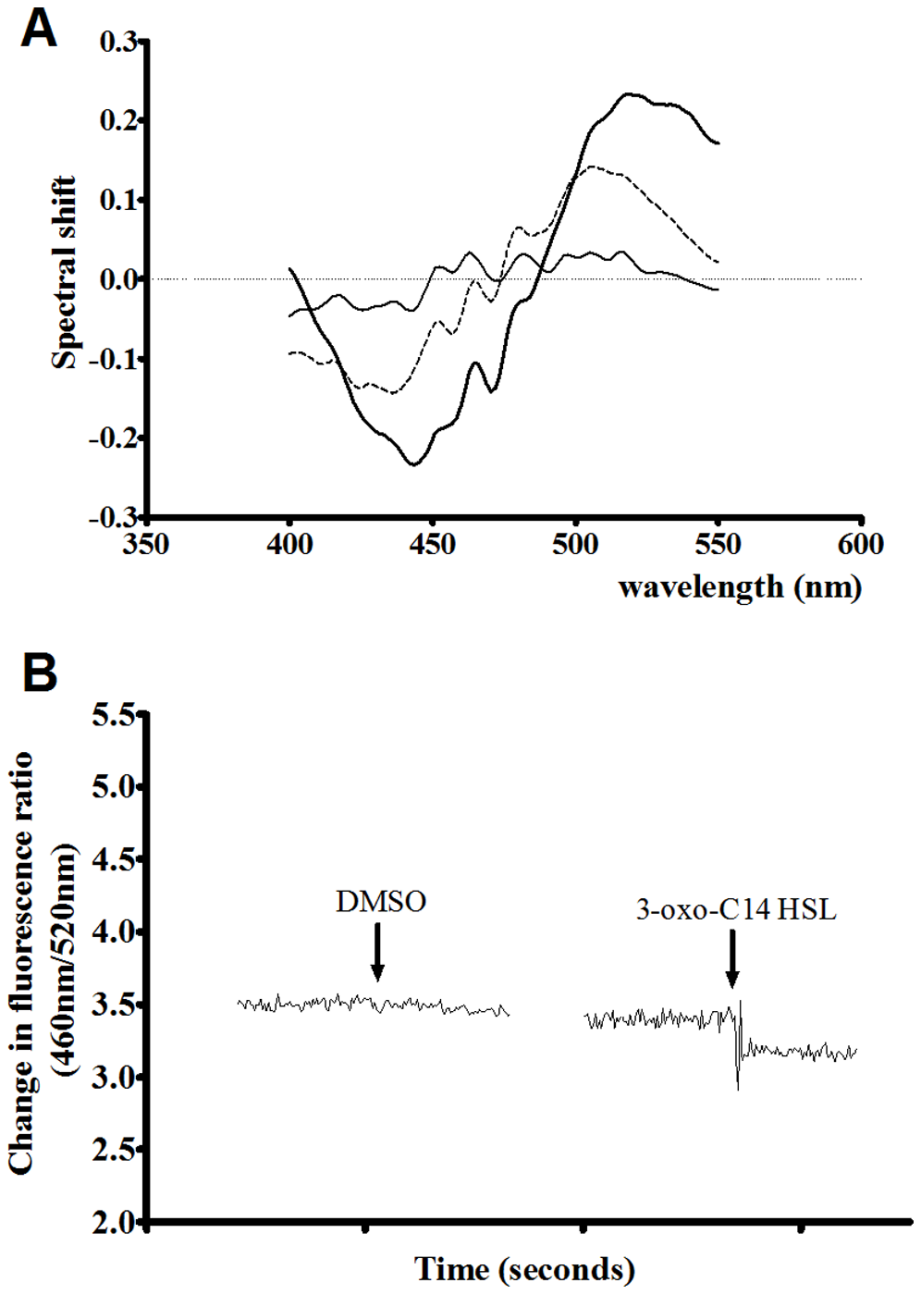
The interactions of AHLs with lymphocyte membranes perturbs the membrane dipole potential. [A] Fluorescence difference spectra obtained by subtracting the di-8-ANEPPS excitation spectra (λ_em_ = 590 nm) of T Lymphocytes (40,000 cells/ml) from those obtained after cells were exposed to the following QS molecules 3-oxo-C_14_-HSL (thick line), 3-oxo-C_12_-HSL (dashed line) and 3-oxo-C_10_-HSL (thin line). Before subtraction, each spectrum was normalised to the integrated areas so that the difference spectra would reflect only the spectral shifts before each difference spectra was normalised to a DMSO control (not shown). A three point moving average was then applied to reduce noise. [B] A dual wavelength ratiometric measurement of the dipole potential variation in di-8-ANEPPS. In this example additions of 22 µM 3-oxo-C_14_-HSL or equivalent volumes of DMSO were made to T-Lymphocytes (40,000 cells/ml) labeled with 10 µM di-8-ANEPPS. Samples were excited at 460 nm and 520 nm. The fluorescence was read at 590 nm and the ratio *R*(460/520) was calculated (shown). All experiments n = 3.

Figure 6A indicates that addition of 3-oxo-C_14_-HSL, 3-oxo-C_12_-HSL and 3-oxo-C_10_-HSL to this model cell system resulted in a red shift in the excitation spectra of di-8-ANEPPS (all normalized to controls containing the DMSO vector but not the QS) similar to those observed previously with the artificial membrane systems. This suggests that the AHLs used in this study were capable of insertion into the plasma membrane of Jurkat T-lymphocytes leading to changes in the dipole potential.

Resazurin reduction-based cell viability assays were conducted as it is conceivable that treatment of the cells with AHLs may affect viability. It was found however, that no significant effects on cell viability take place at any of the concentrations utilized in our studies (data not shown).

**Figure 7:**
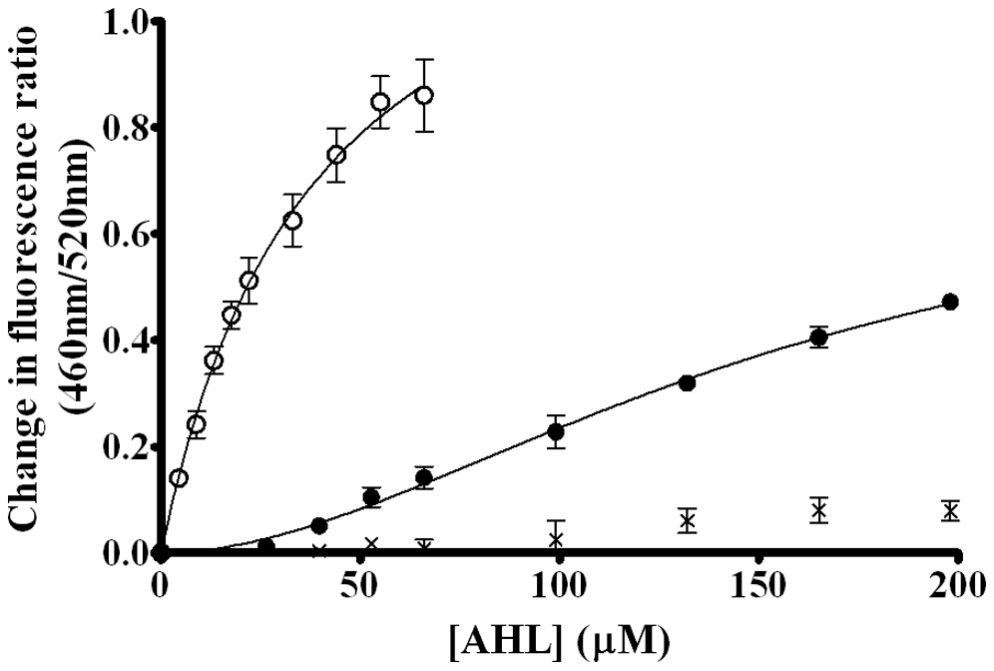
Binding profiles of the interactions of AHLs with Lymphocyte membranes. Binding profiles of 3-oxo-C_14_-HSL (Δ, hyperbolic), 3-oxo-C_12_-HSL (•, sigmoidal) and 3-oxo-C_10_-HSL (×, neither) on titration to di-8-ANEPPS labeled T-Lymphocytes (40,000 cells/ml) at 37°C normalised to DMSO controls. Profiles were fitted to simple hyperbolic and sigmoidal binding models (equations 1 and 2) and F-Tests were used to determine the best fitting model. In each experiment n = 3, ±SEM.

Figure 6B indicates that upon titration of di-8-ANEPPS labeled Jurkat T-lymphocytes with AHLs, a similar decrease in the R(460/520) parameter was observed as for titration with the artificial membrane systems. These data were plotted and fitted as before and are shown in Figure 7. It was found that 3-oxo-C_14_-HSL exhibited a strong interaction with the cell membrane with both a greater binding capacity (1.40±0.11 compared to 0.86±0.08, *p* = 0.02) and dissociation constant significantly less than 3-oxo-C_12_-HSL (39 µM±6 µM compared to 153 µM±38 µM, *p* = 0.04) as determined by two tailed T test. The latter exhibited a more complex binding reaction that was poorly described by Eq. 1. Eq. 2 however was found to be able to describe the binding isotherm and this model indicates that cooperativity appears to be occurring. The cooperativity index (sometimes referred to as the Hill coefficient) for these studies was found to be 1.87±0.27. There are several possible interpretations of this finding, such as that two 3-oxo-C_12_-HSL molecules come together on/in the membrane to promote their respective interaction. There are, however other explanations that accommodate this behavior of which the most likely is that the membrane is modified by the presence of the AHLs and this affects the subsequent binding of further molecules (see below).

3-oxo-C_10_-HSL exhibited only a very slight interaction which fitted neither binding model to a satisfactory degree (R^2^ = 0.57).

**Figure 8:**
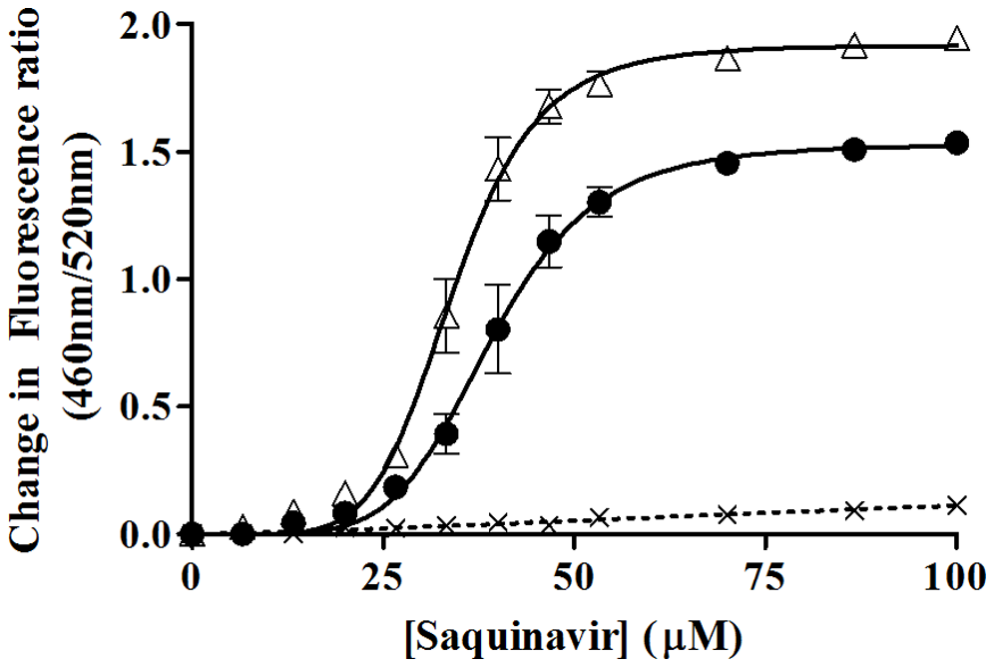
Effect of decreasing membrane dipole potential on membrane receptor conformation. Binding profiles of the P-glycoprotein ligand Saquinavir with T-Lymphocytes (40,000 cells/ml) after pre-treatment with 160 µM 3-oxo-C12 HSL (•) or an equivalent volume of DMSO (final concentration did not exceed 0.5%) (Δ) at 37°C. Data were normalized for saquinavir additions by titrating cells with equivalent volumes of DMSO (×, dashed lines). Profiles were fitted to simple hyperbolic and sigmoidal binding models (equations 1 and 2) and F-Tests were used to determine the best fitting model (sigmoidal for 3-oxo-C_12_ HSL and DMSO pre-treatments). In each experiment n = 3, ±SEM.

Figure 8 indicates that pre-treatment of Jurkat T-lymphocytes with 160 µM 3-oxo-C_12_ HSL caused a significant reduction in the B_max_ of P-glycoprotein (P-gp) for Saquinavir, in comparison to cells treated with equivalent volumes of 0.5% DMSO solvent (1.53±0.04 compared to 1.92±0.03, *p* = 0.002) in addition to a significant increase in K_d_ (39 µM±1 µM compared to 34 µM±1 uM, *p* = 0.007). A slight but insignificant decrease in binding cooperativity was also observed. Subsequent work has shown that pre-treatment of lymphocytes with saquinavir has no significant influence on the interaction of 3-oxo-C_12_ HSL with Lymphocytes (Data not shown). These findings may therefore suggest that 3-oxo-C_12_ HSL mediated lymphocyte membrane dipole potential modulation has indirectly acted to change the activity of P-glycoprotein.

## Discussion

### The Interactions of AHLs with Artificial Membrane Systems

The present study outlines the interactions between long chain AHLs and a number of membrane types including live cells from which it is possible to draw several important conclusions. First 3-oxo-C_14_- HSL, 3-oxo-C_12_-HSL and 3-oxo-C_10_-HSL as shown in Figure 4, were all observed to interact with PC_100%_ and PC_70%_Cholesterol_30%_ artificial membrane systems. These model membranes are taken to represent the most abundant lipid types found in human cell types; their interaction profiles were found to correspond to simple hyperbolic (saturatable) binding models (Figures 3 & 4). Based on the well characterized responses of the fluorescent probe we utilize in this study [30], [31] we are able to conclude from our observations that AHLs are capable of direct insertion into biological membranes in the micromolar concentration range.

### The Interaction of AHLs with Cell Membranes

One virtue of using the di-8-ANEPPS probe is that it may also be implemented with live cells as well as with our model membrane systems. Analogous studies with T cells (Figure 7), therefore, were found to indicate that all three AHLs are also capable of insertion into the cell membrane in the micromolar concentration range.

Previous work examining the immunomodulatory activity of AHLs has suggested that insertion into T-lymphocyte membranes is unlikely to be responsible for immunosuppressive effects of 3-oxo-C_12_ HSL at concentrations of less than 10 µM [20]. This finding is corroborated by this study which suggests little membrane association of 3-oxo-C_12_-HSL with T-Lymphocytes at concentrations less than 30 µM. Above this concentration, however, AHLs appear to have the ability to become inserted into membranes. These findings are also in agreement with suggestions that 3-oxo-C_12_-HSL acts via multiple signaling pathways [1].

The interaction of 3-oxo-C_12_-HSL with T-Lymphocytes was found to be characterized most closely by a cooperative binding mechanism. This suggests that two molecules may come together on/in the membrane resulting in a fruitful binding/insertion complex. As this effect was not observed in the artificial membrane systems this observation implies that either 3-oxo-C_12_ HSL acts to modify the more complex cell membrane which effects the interaction of additional levels of 3-oxo-C_12_ HSL or that there exists an as yet unidentified 3-oxo-C_12_ HSL receptor on the cell surface. The former possibility is less likely as the cell membranes possess both PC and cholesterol at about the levels we use in this study. The latter possibility however, is consistent with previous suggestions of the existence of a membrane receptor system for AHLs. To date the putative eukaryotic receptors identified (PPARs and calprotectin) [25], [26] are both intracellular however the existence of a membrane receptor has been suggested by Shiner *et al.* [32] although its identity or nature has so far remained elusive. Our present work therefore constitutes support for the existence of a eukaryotic AHL membrane receptor which is active at concentrations up to the micromolar range.

The concentrations of AHLs reported to influence membrane dipole potential in this study are slightly greater than those reported to cause significant inhibition of immune cell proliferation [18]. One possible suggestion that reconciles this observation is that the immunosuppressive activity of AHLs is predominantly receptor mediated. The observation that the d-isomer of 3-oxo-C_12_ HSL retains substantial immunomodulatory activity [18], however, implies that indirect membrane interactions also play an important role in the activity of these molecules. In addition, Kaufmann et al. [33] reported more recently that many AHLs are capable of decomposing to form substantial quantities of tetramic acid breakdown products over the prolonged incubation times used (24 to 48 hours in many proliferation assays). The interactions of these tetramic acid breakdown products are as yet poorly understood and may offer an additional explanation for the greater than expected immunosuppressive activity of these molecules.

### The Effects of AHL chain length on the Interaction with Membranes

The AHL acyl chain length was also studied with an increasing chain length observed to augment significantly the membrane binding affinity in both artificial and T-Lymphocyte membrane systems. This behavior was anticipated as an increasing AHL acyl chain length raises the molecular lipophilicity as indicated by the projected octanol/water partition coefficient (Table 1). This parameter is known as Log P and often used by developmental pharmacologists to predict the lipophilicity of drug molecules which has an important bearing on their bioavailability [34]. Classification of the LogP of the AHLs is of interest as it has been previously reported that increasing AHL acyl chain length from C_8_ to C_14_ increased its immunomodulatory activity in the micromolar range [18] and this was found to negatively correlate with LogP (Figure 2). Overall these studies provides further evidence that the membrane interactions of AHLs may play an important role in inter-kingdom signaling including the immune modulatory effects of AHLs.

Figure 5 indicates that modification of the acyl chain to introduce a negative charge (COOH-3-oxo-C_12_ HSL, projected pKa = 4.78±0.1 calculated using ACD/pK_a_ algorithm) significantly reduces the ability of the compound to insert into artificial membranes and modulate the dipole potential. This result was predicted as the LogP of COOH-3-oxo-C_12_ HSL is substantially lower than 3-oxo-C_12_ HSL (Table 1) and provides evidence that changes in the di-8-anepps spectra observed occurred as a result dipole potential modulation, when AHLs inserted into membranes.

### The Interaction of AHLs with Membrane Microdomains

A further sophistication to the systems that we investigate in the present study includes preparation of membranes that we have previously characterized and are known to exhibit microdomains [35]. Such microdomains are rich in cholesterol and appear to be similar to cellular structures known as membrane rafts [36]. The presence of cholesterol containing microdomains on titration of artificial membrane systems with 3-oxo-C_12_-HSL and 3-oxo-C_14_-HSL was observed to increase significantly the saturation point in comparison to membranes that were made up exclusively of phosphatidylcholine. This suggests that the AHLs used in this study may accumulate in cholesterol containing microdomains which leads to a decrease in the membrane dipole potential. The view that membrane microdomain localisation modulates the behavior of receptor signaling is held by research groups in addition to our own [35], [37] and offers an interesting new possibility regarding the activity of receptor controlled signaling systems.

There is a growing body of evidence suggesting that the dipole potential, which is higher in cholesterol rich microdomains than the surrounding disordered membrane, is capable of modulating membrane protein structure which may have implications for raft associated cell signaling [29], [30], [38], [39]. The decrease in membrane dipole potential observed in this study could therefore stimulate the indirect activation of signaling pathways by indirectly instigating a conformational change in transmembrane receptor structure. This principle could apply to both bacterial and eukaryotic organisms and also offers an explanation as to why a primary AHL site of action has so far remained elusive.

### Towards Defining Mechanisms for Inter-kingdom Signaling; Modulation of the Properties of a Membrane Protein Receptor System by AHL-dependent Changes of the Membrane Dipole Potential

The final section of this study is directed towards defining a mechanism by which AHLs may elicit an inter-kingdom signaling process. In other words we seek to demonstrate how a prospective host cell (e.g. T cells) system may respond to the presence of AHLs at appropriate concentrations as an illustration of how such mechanisms may operate. In the present case we take advantage of the fact that the binding of Saquinavir, a HIV-1 protease inhibitor to the membrane protein P-gp is influenced by changes in the membrane dipole potential [29], [40]. The activity of this protein has been shown to have a dependence on the lipidic content of the membrane, particularly the sterol content [41] and appears to be microdomain associated [42], [43]. Previously it has been shown that modification of the membrane dipole potential, through manipulation of sterol concentrations, can influence the activity of P-gp [29]. In the present paper we have shown that pre-treatment of T-lymphocytes with micromolar concentrations of 3-oxo-C_12_ HSL causes a significant reduction in the binding capacity of saquinavir and increase in dissociation constant. These findings are consistent with our previous work (see Asawakarn *et al.* [29]) that indicated that the membrane dipole potential plays an important role in modulating ligand-membrane interactions. We demonstrated that disruption of cholesterol containing microdomains resulted in a decrease in the binding capacity of P-gp for saquinavir. As a similar trend was observed in the present work, this provides further evidence to support the hypothesis that on insertion into membranes, AHLs may act to disrupt cholesterol containing membrane microdomains. This could have important consequences on the activity of a plethora of raft-dependant membrane proteins by no means limited to P-gp [44], although it is interesting that P-gp activity is also associated with anti-microbial therapy.

P-gp belongs to the ABC-transporter protein superfamily and is known to play a crucial role in the development of multiple-drug resistance (MDR) [45]. The ability of AHLs to modulate PgP activity would be advantageous to the bacteria and increase the immune systems susceptibility to cytotoxic agents also released by the pathogen. In addition, as MDR poses a significant problem for the treatment of conditions including cancer, the search for potential inhibitors has been intensive [45], [46], [47]. Our present paper provides evidence of a membrane based mechanism of action of AHLs on immune cells and suggests that through their membrane interactions can act to modulate P-gp activity. AHLs or their derivatives may therefore provide a novel drug template family for the prevention of MDR.

The concentration of AHLs required to modulate immune cell function in this way suggest that only cells in close proximity to the bacterial biofilm would be subject to immune modulation through these processes. This observation is in agreement with predictions by Teplitski et al 2010 [48] who suggests the existence of such an AHL gradient, up to micromolar concentrations under physiological conditions. This could be of evolutionary advantage to *P. aeruginosa* as under this system only immune cells which pose a direct threat to the bacterial population are affected, allowing more remote immune cells to retain their function, potentially eliminating pathogens which would otherwise take advantage of a compromised immune system, developing into secondary infections which would compete with *P. aeruginosa*.

Finally Chhabra *et al.* [18] have suggested that insertion of AHLs into T-lymphocyte membranes causes immunosupression through the inhibition of immunological synapse formation. Membrane microdomains have been shown to play an important role in the formation of the immunological synapse [49]. The insertion of AHLs into biological membranes observed in this study and the associated decrease in dipole potential could be interpreted as evidence that this is the case.

## Materials and Methods

### Reagents

Egg phosphatidylcholine (PC) was supplied by Lipid Products (UK). Di-8-ANEPPS was supplied by Invitrogen, UK. Saquinavir was supplied by Roche (UK). AHLs were synthesized as described previously [18], [50]. Tissue culture reagents, cholesterol and all other reagents were supplied at the highest purity available by Sigma Aldrich (Poole, UK).

### Membrane preparation and labeling with DI-8-ANEPPS

This technique is outlined more comprehensively in [29]. Briefly, PC_100%_ and PC_70%_Cholesterol_30%_ (molar ratios) were dissolved in chloroform before drying under a stream of oxygen-free nitrogen gas by rotary evaporation until a thin film was formed. The lipid film was rehydrated with 280 mM sucrose, 10 mM Tris, pH 7.4 The resulting multilamellar solution was freeze-thawed 5 times in liquid nitrogen and finally extruded 10 times through polycarbonate filters with pores 200 nm in diameter (Nucleopore Corp., Pleasanton, USA) using an extruder (Lipex Biomembranes Inc., Vancouver, Canada) according to the extrusion procedure [51]. This resulted in a monodisperse, unilamellar suspension of phospholipid vesicles. These were labeled exclusively in the outer bilayer leaflet with Di-8-ANNEPS. Here the phospholipid vesicles were incubated for at least 1.5 hours at 37°C in the dark in the presence of Di-8-ANEPPS dissolved in ethanol.

### Cell viability

An alamarBlue (Invitrogen; Carlsbad, CA) resazurin reduction ass*ay* was used to measure of the number of viable and proliferating cells and was conducted according to the manufacturer's instructions. After incubation with AHLs compounds, 10% alamarBlue was added to each well. Cells were incubated for 4 h in a 5% CO_2_ incubator at 37°C. A negative control containing culture medium and alamarBlue reagent without cells was included. Fluorescence measurements were made by excitation at 530–560 nm and measuring emission at 590 using a LS-55 plate reader (Perkin Elmer, MA, USA).

### Labeling of Jurkat T-Lymphocytes with Di-8-ANEPPS

The cells were cultured in RPMI 1640 medium supplemented with 10% foetal bovine serum, L-glutamine (0.02M), penicillin (100units/ml) and streptomycin (100 mg/ml) and maintained at 37°C and 5% CO_2_. Cells were counted using a trypan blue exclusion assay before harvesting by centrifugation at 300 g for 5 minutes. Cells were then labeled with Di-8-ANEPPS according to the methods outlined by Asawakarn *et al*. [29] as follows: 0.5 µM Di-8-ANEPPS was added to a suspension containing 1×10^6^ cells per ml^−1^ and incubated for 1.5 h at 37°C.

### Fluorescence measurements

Fluorescence time courses were undertaken by adding desired amounts of an experimental reagent under study to suspensions of cells or phospholipid vesicles (400 µM lipid or 40,000 cells ml^−1^) on a Fluromax-4 model spectrofluorimeter (HORIBA Jobin Yvon Thermo Electron, UK). Di-8-ANEPPS spectra were obtained by exciting the samples at 460 nm and 520 nm and measuring the emission ratio at 590 nm [29], [52]. The contribution of the dilution effect to the fluorescence signal was corrected by using equivalent liposome or cell suspensions and adding equivalent amounts of DMSO solvent (final concentration did not exceed 0.6%). Any changes in fluorescence signal were then subtracted from those obtained with the quorum compounds. These data were fitted to Equations 1 & 2 in order to determine the best description of the molecular process. The model which best fit these data was determined using an Extra sum-of-squares F-Test.
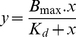
(1)

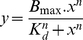
(2)


Where *y* is the observed signal, B_max_ is the 100% ligand binding capacity (fluorescent units), K_d_ is the affinity of the QS compound for the membrane in concentration units and n represents the hill coefficient, i.e. as an index of cooperativity. Unless otherwise stated all experiments are reported from at least 3 replicates ie. n = 3.
